# Workplace factors can predict the stress levels of healthcare workers during the COVID-19 pandemic: First interim results of a multicenter follow-up study

**DOI:** 10.3389/fpubh.2022.1002927

**Published:** 2022-11-01

**Authors:** Na-na Xiong, Teng-teng Fan, Rainer Leonhart, Kurt Fritzsche, Qi Liu, Lei Luo, Barbara Stein, Christiane Waller, Mingjin Huang, Markus M. Müller, The Cope-Corona Working Group

**Affiliations:** ^1^Peking University Sixth Hospital, Peking University Institute of Mental Health, NHC Key Laboratory of Mental Health (Peking University), National Clinical Research Centre for Mental Disorders (Peking University Sixth Hospital), Peking University, Beijing, China; ^2^Department for Social Psychology and Methodology, Institute of Psychology, University of Freiburg, Freiburg, Germany; ^3^Department of Psychosomatic Medicine and Psychotherapy, University Medical Centre Freiburg, Freiburg, Germany; ^4^Department of Psychosomatic Medicine and Psychotherapy, Nuremberg General Hospital, Paracelsus Medical University, Nuremberg, Germany; ^5^The Third Hospital of Mianyang, Sichuan Mental Health Center, Mianyang, China; ^6^Social and Organizational Psychology, Catholic University Eichstätt-Ingolstadt, Eichstätt, Germany

**Keywords:** health care workers, mental health, organizational factors, stress, workplace factors

## Abstract

**Background:**

Research is lacking on the long-term influence of workplace factors on the mental health of health care workers during the COVID-19 pandemic.

**Methods:**

We distributed two online surveys to health care workers between May and October 2020 (T1) and between February and April 2021 (T2). Perceived stress, coronavirus-related risks, and workplace factors were measured *via* self-report questionnaires at both time points. We conducted hierarchical linear regression to investigate the predictive factors for high stress.

**Results:**

A total of 2,110 participants from seven countries and 4,240 participants from nine countries were enrolled at T1 and T2, respectively. Among them, 612 participated in both surveys. We called this cohort T1 + T2. High stress was reported in 53.8 and 61.6% of participants at T1 and T2, respectively. In cohort T1 + T2, compared with the baseline, the level of stress rose significantly (6.0 ± 2.9 vs. 6.4 ± 3.1), as did health/safety in the workplace (3.9 ± 0.8 vs. 4.2 ± 0.7). Unfortunately, we did not detect any significant difference concerning support in the workplace. Among all factors at baseline, being older than 35 [β (95% CI) = −0.92 (−1.45, −0.40)], support [−0.80 (−1.29, −0.32)], and health/safety in the workplace [−0.33 (−0.65, −0.01)] were independent protective factors, while a positive history of mental disorders [0.81 (0.26, 1.37)] and rejection in private life [0.86 (0.48, 1.25)] were risk factors for high stress at T2.

**Conclusion:**

To relieve the high stress of health care workers, organizational-level approaches should be implemented, especially measures designed to enhance support, health/safety in the workplace, and to reduce the rejection of the public.

## Introduction

According to the World Health Organization (WHO), as of 30 November 2021, the coronavirus disease 2019 (COVID-19) pandemic has resulted in 261,435,768 confirmed cases globally, including 5,207,634 deaths. It has posed an unprecedented threat to the entire world (1).

Given the human-to-human transmission of the coronavirus, frontline health care workers (HCWs) have been at high risk of becoming infected through close contact with COVID-19 patients. In addition, when the number of patients exploded and medical resources were insufficient, HCWs were under tremendous stress, both physically and psychologically (2). The relative shortage of personal protective equipment (PPE) in the very early stages of the pandemic, overwork, frustration, discrimination, isolation, and worrying about family members made the challenge more difficult (3). Therefore, the mental health of HCWs has been negatively affected. A meta-analysis showed that the pooled prevalence rates of moderate to severe post-traumatic stress symptoms, anxiety, depression, and sleep disturbances among HCWs in China were as high as 27, 17, 15, and 15%, respectively during the COVID-19 pandemic. Among them, frontline HCWs, women, nurses, and those working in Wuhan, China reported more severe degrees of mental health symptoms (4). In addition, a high prevalence of professional burnout was detected (5–7).

To mitigate the mental health problems and stress of HCWs, multifaceted interventions have been proposed and adopted. On the individual level, psychological support has been provided through a variety of methods to help heavily burdened HCWs, including hotline services, psychological counseling, online platforms with psychological self-help information, and support groups (8, 9). At the organizational level, harmful workplace factors have been identified and improved to prevent the deterioration of the mental health and wellbeing of HCWs. Some work-related risk factors have been investigated and discussed more frequently, including high COVID-19 exposure and heavy workload (10). Therefore, adequate PPE, shorter work shifts, and convenient accommodations and diet have been recommended to buffer against the negative impact of stress (5, 11, 12). However, other workplace factors—such as support and cohesion in the workplace and rejection or discrimination toward HCWs due to the risk of contagion—have been far less discussed. Based on previous experiences, if handled improperly, these factors can lead to poor communication, impaired trust, and weakened teamwork. Hence, teams that were newly formed in the course of coronavirus-related restructuring or that have come into conflict situations due to stress overload should pay special attention to these factors. Moreover, most studies concerning COVID-19 have been cross-sectional, and the long-term effect of workplace factors remains unknown.

In sum, to provide evidence for governments and policy-makers, the association between workplace factors and the stress of HCWs during the COVID-19 pandemic needs to be investigated in a large-scale sample over the long term. Thus, as part of the Cope-Corona project, we aimed to evaluate the level of stress, coronavirus-related risks (the frequency of contact with COVID-19 patients and self-perceived risk), and workplace factors (support in the workplace, health/safety in the workplace, and rejection in private life due to work) of HCWs from multiple countries and how they have changed during the COVID-19 pandemic, as well as to identify predictive factors of a high level of stress.

## Materials and methods

### Study design

This study was part of the Cope-Corona project, which aims to investigate how medical staff have handled the coronavirus pandemic, and to examine their resources and coping strategies. In addition to the variables reported here, further scales measuring individual resources and psychological reactions to the pandemic will be reported and analyzed in subsequent papers.

The working group was founded based on the European Association for Psychosomatic Medicine (EAPM), along with Paracelsus Medical University, Nuremberg General Hospital, and Catholic University Eichstätt-Ingolstadt, led by C. Waller serving as the principal investigator. All members of EAPM were informed about the research initiative and asked to participate. The whole project was designed to be carried out at three points in time: T1 between June and October 2020, during the first phase of the pandemic; T2 between February and April 2021, during a possible second peak; and T3 in Spring 2022. This process resulted in a group of partners situated in Ireland, Andorra, Spain, Germany, Italy, Romania, and Iran for T1. At T2, partners in Poland and China joined the working group.

We designed the study as an online survey using the Qualtrics survey tool (https://www.qualtrics.com). The survey was made available in German, English, Spanish, Catalan, Italian, Romanian, and Farsi. Additional versions in Polish and Chinese were available at T2. The Qualtrics tool reads the language settings from the user's browser and presents the adequate language accordingly.

The survey was fully anonymized. We did not gather any IP addresses or geographic data. Subjects were asked to give a self-generated identification code to match subjects at the different assessment points in time.

We obtained ethical approval from the Institutional Review Board of Paracelsus Medical University, General Hospital Nuremberg (No. IRB-2020-017) and from each study center. All participants received full disclosure and provided informed consent.

### Participants

All adult (≥18 years old) employees of the hospitals and their subcontractors—including medical doctors, nurses, medical-technical personnel, psychologists, medical students, administrative workers, and researchers—were asked to participate in the survey. Otherwise, there were no exclusion criteria. To ensure the validity of the responses, the inclusion criterion was a response to at least 50% of the questions. This means that we excluded participants from the analysis who answered less than 50% of the questions. The 50% rule was agreed upon at the beginning of the survey by the members of the research group as a heuristic approach to exclude unfinished surveys. All questions were weighted the same. At both measurement time points, all employees were invited to participate in the survey, regardless of their previous participation.

### Instruments

We measured the constructs in the questionnaire using established, validated psychometric scales or with *ad hoc* instruments where appropriate tools were not available. We analyzed all *ad hoc* scales using confirmatory factor analysis (CFA) and tests for internal consistency with satisfying results (for details, see Muller et al., under publication).

Perceived stress. We gauged perceived psychological stress using the Perceived Stress Scale (PSS-4) (13), a self-report tool to evaluate the degree to which individuals feel about controllability and confidence in handling stressful situations in the previous month. The PSS-4 consists of 4 items, with the answers being rated on a 5-point scale (0 = “never”, 1 = “almost never”, 2 = “sometimes”, 3 = “fairly often”, and 4 = “very often”). The psychometric properties of the PSS-4 are acceptable across cultures and countries (14, 15).

Contact with COVID-19 patients. Respondents were asked whether they dealt directly with coronavirus-infected patients or suspected cases in their work. Answers were scaled from 1 = “not at all”, 2 = “rarely”, 3 = “sometimes”, and 4 = “very much”.

Risk perception. At T1, personal risk concerning the coronavirus was measured with three items on 5-point scales, specifying the probability of becoming infected (“extremely improbable” to “extremely probable”), the danger of becoming infected themselves (“completely harmless” to “extremely dangerous”), and concern about infecting people in their personal lives (“very little” to “very much”). At T2, the question about the probability of becoming infected was rated on a 6-point scale, with an additional option of “I have been infected already”.

Health/safety in the workplace. We measured health/safety in the workplace using two items: one was about the availability of PPE, and the second, more general item was “I am confident that I can stay healthy at work”. Both were rated on 5-point scales (1 = “strongly agree”, 2 = “agree”, 3 = “undecided”, 4 = “disagree”, and 5 = “strongly disagree”).

Support in the workplace. We gauged support in the workplace with five items using statements representing the quality of within-team collaboration, cross-team communication, trust in supervisors, recognition from supervisors, and information provided by the hospital. All items used 4-point scales ranging from 1 = “strongly disagree”, 2 = “disagree”, 3 = “agree”, and 4 = “strongly agree”.

Rejection in private life. We measured rejection in private life concerning one”s job at the hospital using two items: one clarifying rejection or hostility experienced in private life, and the other involving support for one's job in private life. Both were measured on 4-point scales (1 = “strongly disagree”, 2 = “disagree”, 3 = “agree”, and 4 = “strongly agree”).

Demographic and occupational variables. We measured job experience in three categories (less than 3 years, 3 to 6 years, and more than 6 years). In addition we examined sex, age, job position at the hospital, and the previous history of mental illnesses.

### Definition of a high level of stress

The stress level was reflected by the sum score of the PSS-4, which ranged from 0 to 16. Higher scores indicate higher levels of stress. However, in previous literature, no cut-off values were established for a high level of stress. In previous studies on the association between perceived stress and cardiovascular disease (16) or peripheral artery disease (17), a score of 6 has been adopted based on its distribution within the populations studied. In our study, the 25th, 50th, and 75th percentiles of the PSS-4 scores in both the T1 and T2 samples were 4, 6, and 8, respectively. Hence, we employed a PSS-4 score of 6 to categorize participants with high levels of self-perceived stress.

### Statistical methods

As reported in the instrument section, we provided an additional option of “I have been infected already” for the question on the probability of becoming infected at T2, which should have been coded as “6”. As a result, 359 (8.5%) participants chose this option. However, at T1, the question was assessed using a 5-point scale ranging from “1” to “5”. Hence, at T2, a sum score of this construct could lead to a higher estimation of the level of risk perception. To solve this problem, when establishing risk perception at T2, we recoded the option of “I have been infected already” as an invalid value, and excluded participants who chose this option from the analysis. The missing values of other variables were less than 1% and replaced by the linear interpolation method.

For continuous variables, we used an independent samples *t*-test to determine the difference between participants who did and did not have high levels of stress, and the paired samples *t*-test to compare measurements at the two time points. To estimate the effect size, we computed Cohen's d accordingly. The χ2 test was used for categorical variables, and the Bonferroni method was adopted for multiple comparisons. A *p* value of less than 0.05 (two-tailed) was considered significant. We performed hierarchical linear regression analysis to investigate the predictive factors of high stress levels. We entered coronavirus-related risks (both the frequency of contact with COVID-19 patients and self-perceived risk), workplace factors (support in the workplace, health/safety in the workplace, and rejection in private life due to work), and demographic variables that showed a univariate relationship with high levels of stress into the model. We adopted the stepwise method, with a *p* value of less than 0.05 to enter and less than 0.10 required to stay in the model. Statistical analyses were performed with IBM SPSS Statistics, version 24.0.

## Results

### Demographic and occupational characteristics

**Figure 1:**
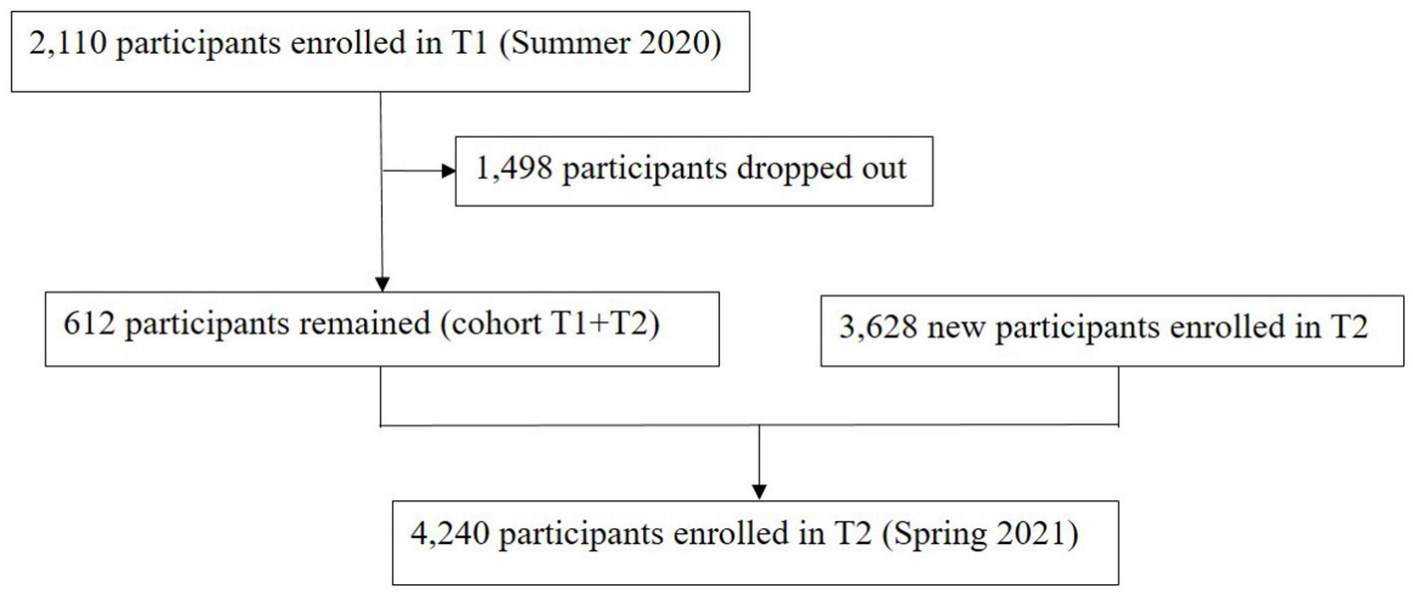
Study flow diagram.

As presented in Figure 1, 2,110 and 4,240 participants were enrolled at T1 and T2, respectively. Of these HCWs, 612 participated in the cohort T1+T2.

The detailed demographic and occupational characteristics are shown in Table 1. At both T1 and T2, the percentages of female and middle-aged HCWs, nurses and doctors, and HCWs with more than 6 years of experience were higher. At T1, most HCWs were from Nuremberg, Germany (63.4%), while at T2, most of them were from three centers in Spain (31.3%) and Nuremberg (27.0%), Germany and two centers in China (12.9%) and Wroclaw in Poland (12.9%), respectively. Besides, 14.6 and 15.5% health professionals reported a positive history of mental disorders in T1 and T2, respectively.

**Table 1:** Demographic and occupational characteristics of HCWs at T1 (*n* = 2,110) and T2 (*n* = 4,240).

	**Percentages in T1** ^*^					**Percentages in T2** ^*^				
**Variables**	**Total (*n =* 2110)**	**Highly stressed (*n =* 1136)**	**Not-highly stressed (*n =* 974)**	**χ^2^**	***p***	**Total (*n =* 4240)**	**Highly stressed (*n =* 2610)**	**Not-highly stressed (*n =* 1630)**	**χ^2^**	***p***
Female	73.5; 100	77.2; 56.6	69.1; 43.4	17.7	**< 0.001**	77.8; 100	80.0; 63.4	74.3; 36.6	18.9	**< 0.001**
Age groups				22.7	**< 0.001**				53.3	**< 0.001**
< 26 years old	12.3; 100	15.0; 65.4^1^	9.2; 34.6			13.0; 100	15.0; 71.1^1^	9.8; 28.9		
26–35 years old	22.5; 100	23.9; 57.3^1, 2^	20.8; 42.7			27.9; 100	29.8; 65.7^1^, ^2^	24.9; 34.3		
36–45 years old	21.9; 100	20.8; 51.1^2^	23.2; 48.9			21.7; 100	21.2; 60.2^2, 3^	22.5; 39.8		
46–55 years old	26.0; 100	24.5; 50.6^2^	27.8; 49.4			22.7; 100	21.0; 56.8^3^	25.5; 43.2		
>56 years old	17.3; 100	15.8; 49.5^2^	18.9; 50.5			14.6; 100	13.0; 54.5^3^	17.3; 45.5		
Position				17.4	**0.002**				11.6	**0.021**
Doctor	19.4; 100	16.3; 45.2^2^	23.0; 54.8			19.3; 100	18.1; 57.8^2^	21.2; 42.2		
Nurse	31.9; 100	33.5; 56.5^1^	30.1; 43.5			37.9; 100	39.7; 64.4^1^	35.0; 35.6		
Technician	10.6; 100	11.6; 58.9^1^	9.4; 41.1			9.1; 100	9.2; 61.8^1, 2^	9.1; 38.2		
Administration	18.5; 100	19.3; 56.2^1^	17.5; 43.8			15.8; 100	15.6; 60.9^1, 2^	16.1; 39.1		
Others	19.6; 100	19.3; 53.0^1, 2^	19.9; 47.0			17.9; 100	17.4; 60.0^1, 2^	18.6; 40.0		
Job experience				20.1	**< 0.001**				24.1	**< 0.001**
< 3 years	17.4; 100	20.3; 62.8^1^	14.1; 37.2			18.2; 100	19.8; 67.3^1^	15.5; 32.7		
3–6 years	11.4; 100	12.5; 59.2^1^	10.1; 40.8			14.8; 100	15.9; 66.1^1^	13.1; 33.9		
>6 years	71.2; 100	67.2; 50. 8^2^	75.9; 49.2			67.0; 100	64.2; 59.0^2^	71.5; 41.0		
Center				45.4	**< 0.001**				137.7	**< 0.001**
Andorra	11.7; 100	10.6; 48.8^3, 4^	12.9; 51.2			7.5; 100	4.9; 39.9^3^	11.7; 60.1		
Barcelona/Dexeus/Val d'Hebron (only T2)	5.5; 100	3.7; 36.2^4^	7.6; 63.8			31.3; 100	28.0; 55.1^2^	36.6; 44.9		
Chieti	6.4; 100	8.3; 69.1^1^	4.3; 30.9			2.1; 100	2.6; 76.7^1^	1.3; 23.3		
Cluj	2.5; 100	2.4; 50.9^1, 2, 3, 4^	2.7; 49.1			0.6; 100	0.6; 60.0^1, 2, 3^	0.6; 40.0		
Ireland	5.3; 100	6.4; 65.8^1, 2, 3^	3.9; 34.2			3.3; 100	3.4; 63.8^1, 2^	3.1; 36.2		
Nuremberg	63.4; 100	62.1; 52.7^2, 3^	64.9; 47.3			27.0; 100	28.7; 65.4^1^	24.3; 34.6		
Tehran	5.3; 100	6.6; 67.6^1, 2^	3.7; 32.4			2.9; 100	3.1; 65.3^1, 2^	2.6; 34.7		
Wroclaw (only T2)	-					12.3; 100	14.5; 72.4^1^	8.8; 27.6		
Beijing/Mianyang (both only T2)	-					12.9; 100	14.2; 67.5^1^	10.9; 32.5		
Positive history of mental illnesses	17.4; 100	19.8; 61.0	14.6; 39.0	9.2	**0.002**	19.2; 100	21.5; 68.9	15.5; 31.1	23.1	**< 0.001**

* To make the comparisons between two subgroups clear, the column percentages are presented in the first line, and then row percentages are presented in the second line. The Bonferroni method was adopted for multiple comparisons: Values with ^1^ were significantly higher than values with ^2, 3^ and ^4^, and only values with different superscripts were significantly different from each other. *P* values in bold type indicate significant differences.

With a score of 6 or higher in PSS-4 indicating a high level of stress, we categorized the HCWs into two subgroups. A high stress level was reported in 53.8% (1,136/2,110) and 61.6% (2,610/4,240) of the HCWs at T1 and T2, respectively.

Compared with their non-highly stressed counterparts, highly stressed HCWs had higher rates of positive history of mental disorders, were younger, and had less job experience; more were female but fewer were doctors. In addition, the shares of highly stressed HCWs differed significantly among different centers, as did the change in proportions in the two surveys. For example, at T1, the proportions of HCWs with high stress were significantly higher in Chieti, Italy (69.1%) and Tehran, Iran (67.6%) than in Barcelona and Dexeus, Spain (36.2%) and Andorra (48.4%). At T2, the shares of heavily stressed HCWs increased the most in Spain and Nuremberg, Germany, and the percentages rose to as high as 76.7% in Chieti, Italy. A large number of HCWs from new centers in T2, i.e., Poland and China, were also under high stress.

### Stress, coronavirus-related risks, and workplace factors

At T1, 36.6% of HCWs reported having much or quite a lot of contact with patients with confirmed or suspected COVID-19. At T2, this amount increased to 47.1%. In addition, 359 (8.5%) HCWs reported that they had already been infected with COVID-19.

As presented in Table 2, compared with not-highly stressed participants, those with high stress perceived significantly higher risks associated with the coronavirus at both time points. However, at T1, we did not find any difference between them regarding the frequency of contact with patients with COVID-19, and we noted only a marginal difference at T2. Concerning workplace factors, HCWs with a high level of stress experienced significantly lower support, less health/safety in the workplace, as well as significantly higher rejection in private life due to work at both T1 and T2.

**Table 2:** Stress, coronavirus-related risks, and workplace factors of HCWs at T1 (*n* = 2,110) and T2 (*n* = 4,240).

	**T1**						**T2**					
**Variables**	**Total (*n =* 2,110)**	**Highly stressed (*n =* 1,136)**	**Not-highly stressed (*n =* 974)**	**χ2/ t**	***p***	**Cohen”s d**	**Total (*n =* 4,240)**	**Highly stressed (*n =* 2,610)**	**Not-highly stressed (*n =* 1,630)**	**χ2/ t**	***p***	**Cohen's d**
Perceived stress	6.0 ± 3.0	8.3 ± 2.1	3.5 ± 1.4	−62.7	< 0.001	2.69	6.4 ± 3.0	8.3 ± 2.0	3.2 ± 1.5	−91.6	< 0.001	2.88
Corona contact (%)				1.4	0.245	-				3.9	0.049	-
Hardly any	63.4	62.2	64.7				52.9	51.72	54.8			
Much	36.6	37.8	35.3				47.1	48.31	45.2			
Risk perception^*^	3.2 ± 0.7	3.4 ± 0.8	3.1 ± 0.7	−8.9	< 0.001	0.40	3.4 ± 0.8	3.4 ± 0.8	3.2 ± 0.8	−7.0	< 0.001	0.25
Support in the workplace	2.8 ± 0.6	2.7 ± 0.6	3.0 ± 0.5	11.4	< 0.001	0.54	2.7 ± 0.7	2.6 ± 0.6	2.9 ± 0.7	14.9	< 0.001	0.46
Health and safety in the workplace	3.8 ± 0.9	3.6 ± 0.9	4.0 ± 0.8	10.2	< 0.001	0.47	3.9 ± 0.8	3.7 ± 0.8	4.1 ± 0.8	10.2	< 0.001	0.50
Rejection in private life	1.8 ± 0.6	2.0 ± 0.7	1.7 ± 0.6	−9.8	< 0.001	0.46	1.9 ± 0.6	2.0 ± 0.6	1.7 ± 0.6	−15.8	< 0.001	0.50

* At T2, a total of 3,745 participants were included in the analysis of this variable, since 359 participants selected the option of “being infected already” and were counted as invalid values; another 136 were missing values. The numbers of participants in the two subgroups were 2,307 and 1,438, respectively.

The distribution of the sum scores of PSS-4 was normal at both surveys. Thus, we performed hierarchical linear regression analysis to explore the independent predictive factors of the sum scores. We selected potential related factors based on the results of univariate analysis. As shown in Table 3, in the first step, we utilized potential demographic and personal characteristics (gender, age, job position, job experience, and history of mental illnesses). Only being older than 35 was an independent protective factor, while being female, and with a positive history of mental disorders were the risk factors for high stress at T1. In the second step, we included risk perception and workplace factors, and all of them remained in the model. Finally, we entered and tested all significant independent predictors from the above steps. As a result, at T1, being older, health/safety in the workplace, and support in the workplace were independent protective factors against high levels of stress, while being female, with a positive history of mental disorders, perceived high risk involved in work, and high rejection due to work were risk factors. The fitting of this model was significant, with 20.9% of the total variance explained. Factors remaining in the hierarchical linear model were the same at T2, with the regression coefficients differing slightly.

**Table 3:** Predictive variables of the sum scores of PSS-4 in the hierarchical linear regression model at T1 (*n* = 2,110) and T2 (*n* = 3,745).

	**T1**					**T2**				
**Positive variables selected**	**Unstandardized β (95% CI)**	**Standardized β**	***p***	**F (d.f.)**	**R–square**	**Unstandardized β (95% CI)**	**Standardized β**	***p***	**F (d.f.)**	**R–square**
**First step**				21.7 (3)	0.031				59.2 (3)	0.040
Positive history of mental illnesses	0.91 (0.57, 1.25)	0.12	< 0.001			0.86 (0.64, 1.09)	0.11	< 0.001		
Age	−0.66 (−0.93, −0.39)	−0.11	< 0.001			−0.37 (−0.44, −0.30)	−0.15	< 0.001		
Gender	0.57 (0.28, 0.86)	0.09	< 0.001			0.44 (0.22, 0.65)	0.06	< 0.001		
**Second step**				125.6 (4)	0.193				142.1 (4)	0.133
Risk perception	0.14 (0.10, 0.18)	0.14	< 0.001			0.31 (0.19, 0.44)	0.08	< 0.001		
Support in the workplace	−0.30 (−0.36, −0.24)	−0.23	< 0.001			−0.52 (−0.67, −0.37)	−0.12	< 0.001		
Health/ safety in the workplace	−0.09 (−0.13, −0.05)	−0.10	< 0.001			−0.61 (−0.73, −0.48)	−0.16	< 0.001		
Rejection in private life	0.23 (0.18, 0.28)	0.19	< 0.001			0.97 (0.82, 1.12)	0.21	< 0.001		
**Third step**				75.7 (7)	0.209				98.7 (7)	0.156
Positive history of mental illnesses	0.77 (0.47, 1.08)	0.10	< 0.001			0.75 (0.52, 0.97)	0.10	< 0.001		
Age	−0.32 (−0.56, −0.07)	−0.05	0.009			−0.26 (−0.33, −0.19)	−0.11	< 0.001		
Gender	0.59 (0.33, 0.85)	0.09	< 0.001			0.33 (0.12, 0.55)	0.05	0.002		
Risk perception	0.59 (0.42, 0.76)	0.14	< 0.001			0.31 (0.19, 0.44)	0.08	< 0.001		
Support in the workplace	−1.20 (−1.43, −0.97)	−0.23	< 0.001			−0.49 (−0.64, −0.35)	−0.10	< 0.001		
Health/ safety in the workplace	−0.29 (−0.46, −0.13)	−0.09	< 0.001			−0.57 (−0.69, −0.44)	−0.15	< 0.001		
Rejection in private life	0.84 (0.65, 1.03)	0.18	< 0.001			0.92 (0.77, 1.07)	0.20	< 0.001		

β, partial regression coefficient. Definition of variables: age group: 1 = equal to or younger than 35, 2 = older than 35; gender: 1 = female, 2 = male.

In T2, 12.9% (548/4,240) participants were from Chinese centers. To further clarify the potential influence of different health care systems and cultural backgrounds on workplace factors, comparisons between HCWs from Western and Chinese centers were carried out concerning the stress, coronavirus-related risks and workplace factors. According to the results (see Supplementary Table 1), even though the perceived stress level was similar between Chinese and Western subjects, the percentage of Chinese HCWs who had frequent contact with patients with COVID-19 was significantly lower. In consistent with this, their risk perception was significantly lower, and the perceived health and safety in the workplace was significantly higher. However, Chinese HCWs reported a significantly lower level of support in the workplace and a higher level of rejection in private life. In a next step, the associated factors of high stress level in T2 were explored within the Chinese subsample. According to the results, only health/safety in the workplace, and rejection in private life due to work could predict a high stress level.

### Changes in stress and predictive factors

A total of 612 out of 2,110 (29.0%) HCWs in T1 completed T2; they constituted cohort T1 + T2. Among all participants at T1, the level of perceived stress (6.0 ± 2.9 vs. 6.0 ± 3.0) did not differ between those in cohort T1+T2 and those not in cohort T1 + T2.

In cohort T1 + T2, compared with the baseline at T1, both the stress level and frequency of contact with COVID-19 patients rose significantly after more than half a year at T2 (see Table 4). Fortunately, health/safety in the workplace increased as well, with a moderate effect size. However, we did not observe a significant difference concerning perceived support in the workplace and rejection in private life due to working in hospitals.

**Table 4:** Change in stress, coronavirus-related risks, and workplace factors of HCWs in cohort T1+T2 (*n* = 612).

	**Baseline (T1)**	**T2**	**t/χ2**	***p***	**Cohen's d**
Perceived stress	6.0 ± 2.9	6.4 ± 3.1	−4.1	< 0.001	0.13
Corona contact (%)			260.0	< 0.001	-
Hardly any	69.3	63.8			
Much	30.7	36.2			
Risk perception^*^	3.2 ± 0.7	3.2 ± 0.7	−2.0	0.051	0.13
Health/safety in the workplace	3.9 ± 0.8	4.2 ± 0.7	−9.0	< 0.001	0.40
Support in the workplace	2.9 ± 0.5	2.8 ± 0.6	1.8	0.069	0.18
Rejection in private life	1.8 ± 0.6	1.8 ± 0.6	0.7	0.478	0.03

* A total of 574 participants were included in the analysis of this variable, since 38 (6.2%) participants selected the option of “being infected already” and were counted as invalid values.

In cohort T1 + T2, correlation analysis showed that among all demographic, occupational, and work-related factors at baseline, a positive history of mental disorders (r = 0.127, *p* < 0.001), age (r = −0.161, *p* < 0.001), job experience (r = −0.127, *p* = 0.001), risk perception (r = 0.114, *p* = 0.003), support in the workplace (r = −0.206, *p* < 0.001), health/safety in the workplace (r = −0.192, *p* < 0.001), and rejection in private life (r = 0.219, *p* < 0.001) were significantly correlated with a high level of perceived stress at T2. Entered into the multivariate linear regression model, similar to the above model at T1 and T2, age (>35 years old) [β (95% CI) = −0.95 (−1.45, −0.41)], support in the workplace [−0.92 (−1.42, −0.43)], and health/safety in the workplace [−0.46 (−0.79, −0.14)] at baseline were protective factors, while a positive history of mental disorders [0.81 (0.26, 1.37)], and rejection in private life at baseline [0.43 (0.11, 0.72)] were risk factors for a high stress level at T2. The model was significant, with 11.3% of the total variance explained.

## Discussion

As the first interim result of the Cope-Corona project, a high level of stress was reported in more than half of the HCWs; this proportion continued to rise in the follow-up study. With longitudinal data, we expanded upon past research by demonstrating that workplace factors (support in the workplace, health/safety in the workplace, and rejection in private life due to work)—instead of coronavirus-related risks—can predict not only the present stress level, but also the stress level after more than half a year during the COVID-19 pandemic.

Similar to our results, previous evidence worldwide has shown that mental problems became pronounced in HCWs during the COVID-19 pandemic, as well as in other viral epidemics. According to a systematic review on the psychological impact of HCWs during large-scale viral outbreaks, the pooled prevalence for acute stress disorder was 40%, followed by anxiety (30%), burnout (28%), depression (24%), and post-traumatic stress disorder (PTSD) (13%) (18). In addition, younger age, being female, and lower levels of specialized training and job experience were associated with those problems. A survey in England included 106 HCWs and found that the median stress (PSS-4) score of the overall cohort was 7 (19). In this survey, the stress level was significantly higher in respondents with pre-existing depression and anxiety. Similarly, a higher level of stress was observed in younger, female HCWs, and HCWs with a previous history of mental disorders, and less job experience and from several study centers in this study. Being younger than 35 years old was also the independent risk factor for a high stress level according to the longitudinal data. The possible explanation could be that younger medical staff were generally less experienced, and were more likely to be sent as the front-line workers. In addition, older medical staff probably have already experienced other viral epidemics occurred in the past 20 years, such as the severe acute respiratory syndrome (SARS) in 2003, the A/H1N1 influenza pandemic in 2009, Middle East respiratory syndrome (MERS) in 2012, and Ebola virus disease in 2014, and thus were better prepared for the outbreak.

However, unlike other studies, the COVID-19-related risks did not seem to play an important role in the elevated stress level in our study. According to our results, the stress level was not associated with the frequency of contact with COVID-19 patients. Even though perceived risk involved in work was associated with a high level of stress, it could not predict the stress level after more than half a year. According to studies about the mental health of HCWs in previous viral epidemic outbreaks, feeling unsafe and a lack of specialized training were adverse factors psychological wellbeing (18). Therefore, a possible explanation for such a phenomenon is that during late phase of the COVID-19, basic organization-wide approaches were already provided by most hospitals, like sufficient protective measures and specialized skills training, and have helped to mitigate the harm of coronavirus-related risks.

Besides, baseline support and health/safety in the workplace have been proved to be protective factors against high stress, while rejection in private life at baseline was a risk factor for a high stress level in our study. In line with our findings, a cross-sectional study in the US indicated that organizational risk factors for adverse psychological effects in HCWs were limited PPE and negative feelings toward one's workplace, e.g., not believing in the values and actions of one's organization and feeling unable to refuse specific organizational demands (20). Another cross-sectional study with 2,527 responses from 41 countries demonstrated that depressive symptoms have been associated with perceived poor workplace support during the COVID-19 pandemic (21). In addition, 3 months after COVID-19 was declared a pandemic, a large sample study in Singapore showed that the burnout threshold was met by more than three quarters of respondents, and that psychological pathology was associated with a lack of safe work environments (5). Therefore, coordinated actions between workers, health teams and health institutions were recommended to be included as part of a comprehensive community care to protect the mental health emergency generated by COVID-19 (22).

Before the COVID-19 pandemic, previous researches have already showed that adverse workplace factors were associated with the likelihood of developing mental health problems among HCWs. For example, based on a structured interview, workplace conflicts that have a negative influence on the mental health of health professionals were classified under three key themes: individual, interpersonal, and organizational factors (23). According to this framework, the sources of organizational conflict predominantly include ambiguity in professional roles or workflows and poor work environments. Organizational-level approaches like regularly reviewing workplace policies and protocols on how to support employees after an occupational traumatic event, ensuring that practice is consistent with established best practice, and offering task-focused skills training have been recommended to protect the mental wellbeing of individuals at high-risk occupations (24).

However, to date, most interventions implemented to relieve the mental health problems of HCWs during the COVID-19 pandemic focused mainly on individual symptoms, but rather the organizational or interpersonal levels (25). For example, the most common intervention was providing individual mental health services, such as psychological counseling (8). Notwithstanding, these interventions might hinder efforts to explore the impact of organizational or systemic factors on adverse mental health outcomes (25). In addition, interventions at the individual level often met with the problem of low acceptance or low interest, as Chen et al. (26) pointed out. In this study, even though medical staff showed signs of psychological distress, they denied having problems and refused psychological help. Workplace-based interventions, such as providing more places to rest and guaranteeing food and daily living supplies, were welcomed (26). In another study in China, a series of workplace-based interventions were provided, including a mandatory 2-week quarantine in the hospital after work shifts in the fever clinic to avoid infecting family members; reasonable adjustments of working hours; the provision of convenient accommodations, diet, and adequate PPE; the help of a labor union; and help for family members when needed (27). Organizational interventions also include clear communication with staff (28) and the implementation of training programmes (29). To solve the problem of workplace violence against health professionals during the coronavirus pandemic, researchers have also recommended hospitals and governments to help to promote the transference of information to patients, to implement appropriate sanctions on the convicts, to promote interpersonal support in professional groups, and to raise public awareness (30). Unfortunately, as our results show, even after more than half a year during the pandemic, support in the workplace did not increase and remained at a low level.

Moreover, the last factor that needs to be discussed is the stigmatization of people working in high-risk environments. Consistent with our findings, a recent study in an Italian hospital showed that stigma was positively correlated with an increased risk of burnout and fatigue in HCWs (31). However, regretfully, we also found that discrimination against HCWs did not decline during the previous year. Hence, efforts to decrease the discrimination and rejection of the public toward not only HCWs, but also patients with a history of COVID-19, should not be neglected.

In addition, given consideration to the potential influence of different health care systems and cultural backgrounds of Western and Chinese centers, further comparisons were compared between them. Results indicated that Chinese HCWs had less contact with COVID-19 patients, a lower level of corona-related risk perception, and a higher level of health and safety in the workplace. Such a low level of risk was consistent with the low infection rate of COVID-19 in China due to the strict prevention and control measures adopted by the government. However, the stress level between Chinese and Western medical staff was comparable, and Chinese HCWs reported a significantly lower level of support in the workplace and a higher level of rejection in private life. Those negative workplace factors could explain the high stress in Chinese medical staff. Therefore, the results further highlighted the importance of approaches designed to improve workplace factors in improving the wellbeings of healthcare workers during the pandemic, despite their different cultural backgrounds, medical systems and epidemic-related policies. However, results from Chinese centers should be interpreted with caution due to the small sample size.

This study has several limitations. First, even though we recruited HCWs from multiple centers in Europe and China, most of our data was based on European centers and the representativeness of this sample was limited. As such, the results should be interpreted with caution concerning HCWs from different countries. In particular, different health care systems and cultural backgrounds could have an influence on the severity of mental stress and the influence of workplace factors. Second, only a small proportion of HCWs participated at both T1 and T2, which led to a lower generalizability of the results from the longitudinal data. Nevertheless, such a proportion has been common for similar online surveys during the pandemic. We did not observe any significant difference between people who participated in T2 (or not) concerning the level of perceived stress. In addition, we verified the multiple linear regression model using both cross-sectional and longitudinal data. Third, we noted different stress levels among different centers. This might be the outcome of the complex interaction of multiple factors, such as the incidence of COVID-19, the availability of medical and personnel resources, and a previous history of mental health problems of HCWs. We tried to explore its influence by comparing subjects from Western and Chinese centers, but more detailed comparison and discussion will be needed in the future research. Lastly, the categories of age and job experience were determined based on a heuristic approach at the beginning of the survey by the members of the research group. A more thorough assessment would be to use validated work experience scales.

In sum, using longitudinal data of HCWs from multiple centers, during the COVID-19 pandemic, the level of perceived stress has been high among HCWs, and kept increasing after more than half a year. In addition, age, a positive history of mental disorders, and workplace factors (support in the workplace, health/safety in the workplace, and rejection in private life due to work)—instead of coronavirus-related risks—can predict not only the present stress level, but also stress level over the long term. Hence, our results highlight the importance of interventions and policies at the organizational level in promoting the mental health of HCWs. Specific measures should be designed to improve support, health/safety in the workplace, as well as to reduce the rejection of the public.

## The Cope-Corona working group

Eva Baillès, Jordi Blanch, Marta Cervera Teluel, Chiara Conti, Padraic J. Dunne, Mihaela Fadgyas Stanculete, Josep Maria Farré, Elena Font, Mireia Forner Puntonet, Kurt Fritzsche, Elena Gayán, Gozie Offiah, Maria Teresa Guagnano, Mingjin Huang, Pol Ibañez Jimenez, Sarah König, Nina Kundinger, Roberta Lanzara, Antonio Lobo, Monika Małecka, Błazej Misiak, Markus Müller, Ali-Akbar Nejatisafa, Amadeu Obach, Gemma Parramon, Tomasz Pawlowski, Josep Maria Peri, Josep Antoni Ramos Quiroga, Ilenia Rosa, Araceli Rousaud, Joanna Rymaszewska, Sara Schuster, Barbara Stein, Dorota Szcześniak, Xavier Torres Mata, Christiane Waller, and Nana Xiong.

## Data availability statement

The raw data supporting the conclusions of this article will be made available by the authors, without undue reservation.

## Ethics statement

The studies involving human participants were reviewed and approved by the Institutional Review Board of Paracelsus Medical University, General Hospital Nuremberg. The patients/participants provided their written informed consent to participate in this study.

## Author contributions

N-nX and T-tF contributed equally to the drafting of the manuscript, primary statistical analysis, and data interpretation. T-tF contributed to the conception of this manuscript and was responsible for it. RL contributed to the statistical analysis and data interpretation. MH, QL, and LL were responsible for different research centers in China and for the review and editing of this manuscript. KF, BS, CW, MM, and members in the Cope-Corona working group contributed to the design of this study, the coordination of the work of multiple study centers, and for the review and editing of this manuscript. All authors read and approved the final manuscript.

## Funding

The research in the Beijing center was funded by the Youth Program of the National Natural Science Foundation of China (T-tF, Grant No. 81901345 and N-nX, and Grant No. 81800482), the project of the National Clinical Research Center for Mental Disorders (T-tF, Grant No. NCRC2021M12 and N-nX, and Grant No. NCRC2021M07), and the National Key Research and Development Project (T-tF, Grant No. 2019YFC0118502).

## Conflict of interest

The authors declare that the research was conducted in the absence of any commercial or financial relationships that could be construed as a potential conflict of interest.

## Publisher's note

All claims expressed in this article are solely those of the authors and do not necessarily represent those of their affiliated organizations, or those of the publisher, the editors and the reviewers. Any product that may be evaluated in this article, or claim that may be made by its manufacturer, is not guaranteed or endorsed by the publisher.
