# Supplementary material for: Workplace factors can predict the stress levels of healthcare workers during the COVID-19 pandemic: First interim results of a multicenter follow-up study
Source: Front Public Health. 2022 Nov 1;10:1002927. doi: 10.3389/fpubh.2022.1002927 (PMC9663923; doi:10.3389/fpubh.2022.1002927)
Supplement: Supplementary file 1 [file Table_1.DOCX]

Supplementary Table 1. Stress, coronavirus-related risks and workplace factors of HCWs from Western and Chinese centers in T2 (n=4240)

| Variables | Total  (n=4240) | Western centers  (n=3692) | Chinese centers  (n=548) | χ^2^/ t | *p* |
| --- | --- | --- | --- | --- | --- |
| Perceived stress | 6.4±3.0 | 6.4±3.1 | 6.4±2.2 | -0.1 | .934 |
| Corona contact (%) |  |  |  | 430.5 | **<.001** |
| Hardly any | 52.9 | 46.7 | 94.2 |  |  |
| Much | 47.1 | 53.3 | 5.8 |  |  |
| Risk perception* | 3.4±0.8 | 3.4±0.7 | 2.8±0.8 | 19.1 | **<.001** |
| Support at the workplace | 2.7±0.7 | 2.8±0.6 | 2.2±0.8 | 22.0 | **<.001** |
| Health and safety in the workplace | 3.9±0.8 | 3.8±0.8 | 4.2±0.7 | -8.6 | **<.001** |
| Rejection in private life | 1.9±0.6 | 1.8±0.6 | 2.3±0.5 | -18.1 | **<.001** |
